# Supplementary material for: Self-assembly of highly ordered micro- and nanoparticle deposits
Source: Nat Commun. 2022 Jun 2;13:3085. doi: 10.1038/s41467-022-30660-6 (PMC9163176; doi:10.1038/s41467-022-30660-6)
Supplement: Supplementary file 1 — Supplementary Information [file 41467_2022_30660_MOESM1_ESM.pdf]

Supplementary Information for:

Self-assembly of highly ordered micro- and nanoparticle deposits

*Hossein Zargartalebi[1,2], S. Hossein Hejazi[3], Amir Sanati-Nezhad[1,2]**

[1] Department of Mechanical and Manufacturing Engineering, University of Calgary, Calgary, Alberta T2N 1N4, Canada
[2]BioMEMS and Bioinspired Microfluidic Laboratory, Department of Biomedical Engineering, University of Calgary, Calgary, Alberta T2N 1N4, Canada.
[3]Department of Chemical and Petroleum Engineering, University of Calgary, Calgary, AB T2N 1N4, Canada
*Corresponding author: A.S.N., amir.sanatinezhad@ucalgary.ca

Contents

Supplementary Note 1. Overview of coffee-ring suppression techniques....................................2

Table S1. Comparing the coffee-ring suppression techniques..2

Supplementary Note 2. Theoretical model of the proposed method..4

Figures..8

Figure S1. Particle deposition morphologies..8

Figure S2. The wettability of the pressure-sensitive adhesive (PSA) and the treated glass slide measured by placing a drop of water on the substrate..9

Figure S3. The topography and roughness of the PSA wall, characterized using atomic force microscopy..9

Figure S4. Non-uniform deposition of CNHs nanoparticles on a glass slide with a contact angle of 32°..10

Figure S5. Reproducibility of the CNH particle coatings formed on gold electrodes under coffee-ring and coffee-ring-free conditions, characterized using an electrochemical sensing method...10

Figure S6. Particle tracing during the liquid film evaporation, characterized using microparticle image velocimetry (μPIV)...11

Figure S7. Deposition of CNHs nanoparticles with different mold patterns.............................12

Figure S8. Deposition of 10 μm polystyrene particles on a glass slide......................................13

Figure S9. Stratification of polystyrene particles (1000 nm and 200 nm in diameter) at the increased concentration of φ_0= 0.1 (compared to the case with φ_0= 0.01 in Figure 4b) and the initial film thickness of approximately H=500 nm..14

Figure S10. 3D confocal imaging of the auto-organization of mixed fine- and microparticles over time...15

Figure S11. The monolayer zone dependency on Pe and H/R...16

References..16

Supplementary Note 1. Overview of coffee-ring suppression techniques

Several strategies and methods have been examined to control the coffee-ring phenomenon. According to the recent reviews[1, 2, 3], the coffee-ring occurrence can be generally suppressed through one of the following approaches: (i) hampering the contact line pinning, (ii) apportioning the edgeward capillary flow, and (iii) impeding the suspended particles being carried to the contact line through the capillary flow. We briefly explain each of the existing approaches, their advantages and shortcomings, which supports highlighting the benefits of the technique proposed in the current work (**Table S1**). For the sake of brevity, we only provide a summary of each technique.

Table S1. Comparing the coffee-ring suppression techniques.

Methodology	Process	Advantages	Disadvantages	Ref.
**Superhydrophobic solid surfaces**	Precipitated polypropylene particles, casting a fine reticulated structure<br>Polymer surfaces that are oleophobic<br>Hydrophobic inorganic solids include oxidized zinc, zinc hydroxide, silane-treated, and etched silicon	Passive method<br>Easy-to-use	The establishment of a superhydrophobic surface is costly<br>Where the droplet contains surface-active materials (for example, in biological samples), there exists strong pinning of the contact line<br>Medium uniformity	4, 5, 6, 7, 8, 9
**Controlling Marangoni flow**	Taking advantage of evaporation as an endothermic phenomenon	Easy-to-use	Marangoni flow due to temperature difference in the water droplet is not strong enough.<br>Active method<br>Low uniformity	10, 11
	Using of volatile fluids	Easy-to-use<br>Passive method	Demands additives (not applicable for several applications)<br>May aggressively affect the performance of the deposited layer<br>Medium uniformity	12
	Locally heating the droplet surface using a laser beam	No need for additives	Applying heat to the substrate may inversely affect the composition of particle-laden drops and suspended particles, and consequently, the	13

			performance of the deposited layer<br>Not applicable for biological samples<br>Active method<br>Low uniformity	
	Rising the substrate temperature	No need for additives	Active method<br>Not applicable for biological samples<br>Low uniformity	14
	Adding different solvents, surfactants, and polymers	Easy-to-use<br>Passive method	Demands additives (not proper for many applications)<br>May aggressively affect the deposited layer performance.<br>Medium uniformity	15, 16, 17, 18, 19, 20
**Electroosmosis**	Using microelectrodes consist of a circular electrode around the rim of the droplet and a point electrode at the center of the droplet to generate a radial electric field	No need for additives	Requires external instrument<br>Demands meticulous manipulating the droplet at the center of the electrode<br>Active method<br>Medium uniformity	21, 22
**Electrowetting**	Applying an electric field between a circular electrode and the particle-laden droplet generates a radial inward electroosmotic flow next to the substrate, which can overcome the edgeward capillary flow and prevent coffee-ring occurrence	Control the movement of particles<br>No need for additives	Demands meticulous manipulating the droplet at the center of the electrode and also induces a slight heating effect<br>Limited to volume fractions below 1% and to particle diameters of 100 nm and larger<br>Active method<br>Medium uniformity	23
**Controlling the shape of suspended particles**	Particle-particle interactions and/or particle-solid-liquid as well as particle-liquid-gas interfaces interactions	No need for any treatments<br>Passive method<br>High uniformity	Restricted to a specific morphology of particles	24
**Adjusting pH of the solution**	DLVO interactions*	Passive method<br>Easy-to-use	Intrusive to the substrate<br>Restricted to solutions with specific pH values<br>Medium uniformity	25

**Adjusting confined geometries**	Pinning-depinning methodology	Patternable<br>High uniformity	Needs external instrument<br>Difficult to control the upper surface distance<br>Active method	26, 27, 28
**Dual droplets**	Spreading wetting a droplet over the supporting droplet	High uniformity<br>Passive method<br>Easy-to-use	Needs additives<br>Invasive	29, 30
**Controlling hydrophobicity**	Chemical treatment using hydrophobic molecules	Easy-to-use<br>Passive Method<br>High uniformity	Needs external instrument (inkjet printing)<br>Needs hydrophobic surface	9, 31, 32, 33
	Physical treatment by changing surface morphology via increasing surface roughness	Easy-to-use<br>Passive method	Needs to change the substrate morphology<br>Needs hydrophobic surface<br>Low uniformity	
**(Present method)**	Combination of a mold with neutral wettability and a hydrophilic surface	Passive method<br>Repeatable<br>Easy-to-use<br>Low cost<br>Scalable<br>Independent from particle shape and liquid characteristics<br>Self-assembly of multi-layer deposition<br>Patterning down to microscale features<br>High uniformity	Requires a shadow mold<br>Requires a hydrophilic surface	Present work

* Boris Derjaguin and Lev Landau, Evert Verwey and Theodoor Overbeek (DLVO)

Supplementary Note 2. Theoretical model of the proposed method

The accumulation of particles depends on the pinning time. When the contact angle pins at the surface, the particles start migrating to the contact line due to the capillary flow. Therefore, the more the pinning time, the higher the accumulation of particles at the contact line. To calculate the pinning time, we begin with calculating the volume of the film inside the mold. Assuming negligible gravitational forces in this work (argued in section 'Zeta potential measurement of nanoparticle suspensions), the volume of the film could be calculated as:

$$V = \begin{cases} \pi R^2 H_2 - \frac{\pi R^3}{3} \frac{(1-sin\theta)^2(2+sin\theta)}{cos^3\theta} & \theta > 90° \\ \pi R^2 H_1 - \frac{\pi R^3}{3} \frac{(1-sin\theta)^2(2+sin\theta)}{cos^3\theta} + \pi R^2 H_2 & \theta < 90° \end{cases} \quad \text{(S1)}$$

Therefore, considering $H_1 = R[(1-sin\theta)/cos\theta]$ for $\theta < 90^{\circ}$, the evaporation rate for the wetting and non-wetting phases can be derived as:

$$dV/dt = \begin{cases} \pi R^2 \frac{dH_2}{dt} - \frac{d}{d\theta}\left[\frac{\pi R^3}{3} \frac{(1-sin\theta)^2(2+sin\theta)}{cos^3\theta}\right]\frac{d\theta}{dt} & \theta > 90° \\ \pi R^2 \frac{dH_2}{dt} + \frac{d}{d\theta}\left[\pi R^3 \frac{1-sin\theta}{cos\theta} - \frac{\pi R^3}{3} \frac{(1-sin\theta)^2(2+sin\theta)}{cos^3\theta}\right]\frac{d\theta}{dt} & \theta < 90° \end{cases} \quad \text{(S2)}$$

Simplifying equation (2) and considering $d\bar{V}/dt = \mathrm{dV/dt} - \pi R^2 \mathrm{d}H_2/\mathrm{dt}$, one can estimate the pinning time by integrating equation (2) between $\theta = \theta_o$ (initial contact angle) and $\theta = \theta_o - \delta\theta$ (the contact angle in which the contact line starts receding). Assuming that the pinning starts at the equilibrium contact angle, the pinning time can be determined as:

$$t_{Pin} = \begin{cases} -\frac{\pi R^3}{d\bar{V}/dt} \frac{\delta\theta}{(1+sin\theta)^2} & \theta > 90° \\ -\frac{\pi R^3}{d\bar{V}/dt} \frac{\delta\theta \, sin\theta}{(1+sin\theta)^2} & \theta < 90° \end{cases} \quad \text{(S3)}$$

Considering the confinement of the liquid film within the cavities of the mold, one can assume $d\bar{V}/dt \approx \pi R^2 \dot{E}$, where $\dot{E}$ is the vertical interface velocity during evaporation. During the contact line pinning, particles migrate to the periphery due to the capillary-driven advective flux, where the advection time (t_{Adv}) is scaled by $R/\dot{E}$. The advection build-up of particles at the contact line is warded off by diffusional backflow, owing to the development of particle concentration gradient. The diffusion time (t_{Diff}) is also scaled by R^2/D. Given the use of almost spherical-shape particles at a uniform temperature and possessing the same kinetic energy in a quiescent fluid, the stokes-Einstein equation is considered for diffusion coefficient (D). Therefore, normalizing the pinning time with $\Delta t = t_{Adv} - t_{Diff}$, knowing that the diffusion scale is much less than advection, and considering the Peclet number as $Pe = \dot{E}H/D$, the pinning time is approximated as:

$$\left|\frac{t_{Pin}}{\Delta t}\right| = \begin{cases} \frac{H}{R}\frac{\delta\theta}{Pe(1+sin\theta)^2} & \theta > 90° \\ \frac{H}{R}\frac{\delta\theta\ sin\theta}{Pe(1+sin\theta)^2} & \theta < 90° \end{cases} \tag{S4}$$

To calculate the normalized pinning time, one needs to estimate $\delta\theta$, which is dependent on the potential energy barrier. Knowing the interface area $A_{int} = 2\pi R^2/(1+sin\theta)$, the associated Gibbs free energy, G, is estimated as:

$$\begin{aligned} G &= \sigma_{LV}A_{int} + A_{wetting}(\sigma_{SL} - \sigma_{SV}) \\ &= \sigma_{LV}\, 2\pi R^2/(1+sin\theta) + (\pi R^2 + 2\pi RH)(\sigma_{SL} - \sigma_{SV}) \end{aligned} \tag{S5}$$

where, $\sigma_{LV}, \sigma_{SL}, and\ \sigma_{SV}$ are the liquid surface free energy, and solid/liquid and solid/vapor interfacial energies, respectively. Implementing Young's equation[34] one can replace $(\sigma_{SL} - \sigma_{SV})$ by -$\sigma_{LV}cos\theta$. For a given volume V, the initial contact angle is θ_o but when the liquid film is out of the thermodynamic equilibrium with $\theta_o\ - \delta\theta$, the excess free energy per unit length, $\delta\tilde{G} = \delta G/2\pi R$ is calculated as:

$$\delta\tilde{G} = \frac{\sigma_{LV}R\delta\theta^2}{4}\left[\frac{4+2sin\theta-2sin^2\theta}{(1+sin\theta)^3} + \left(1+\frac{2H}{R}\right)cos\theta\right] \tag{S6}$$

When the contact line is pinned, $\delta\tilde{G}$ increases due to the fact that $\delta\theta$ physically ascends during evaporation. Whenever the excess free energy exceeds the potential energy barrier (U), the contact line is depinned. The value U is variable according to the chemical and physical heterogeneities of the surface. The value $\delta\theta$ depends on the value U. One reasonable assumption is that increasing the wettability increases the potential energy barrier; therefore, $\delta\theta$ could be estimated as $\delta\theta \approx a/\theta^b$, in which a and b possess positive values. One can consider $a = b = 1$ and calculate the normalized pinning time in equation (4).

The particles impinge to the interface with a flux of $F_{impinge} = -c\dot{E}$, where c is the concentration of the particles at the interface. Hence, the impinging flux is estimated as:

$$F_{impinge} = -\frac{m_p}{V_{int}}\dot{E} \tag{S7}$$

where m_p and V_{int} are the particle mass and fluid volume at the interface control volume, respectively, and can be defined as:

$$m_p = \rho_p N_A 4/3\pi r_p^3 \quad \text{and} \quad V_{int} = A_{int}\delta \tag{S8}$$

where ρ_p and r_p are the particle density and radius of the particles, respectively. N_A denotes the number of particles at the interface, and δ is the thickness of the interface control volume. The impinging flux is evaluated as:

$$F_{impinge} = -\frac{\rho_p N_A 4/3\pi r_p^3}{A_{int}\delta}\dot{E} \tag{S9}$$

Assuming δ value is equal to $2r_p$, the normalized impinging flux, $\bar{F}_{impinge}$ is calculated as:

$$\bar{F}_{impinge} = \frac{1}{3}\frac{N_A r_p^2}{R^2}(1 + sin\theta) \tag{S10}$$

The saturated monolayer deposition of particles is achieved when $N_A r_p^2 = R^2$. For $N_A r_p^2 < R^2$ or $N_A r_p^2 > R^2$ based on the contact angle, one might experience an unsaturated monolayer regime or a particle accumulation regime, respectively.

Figures

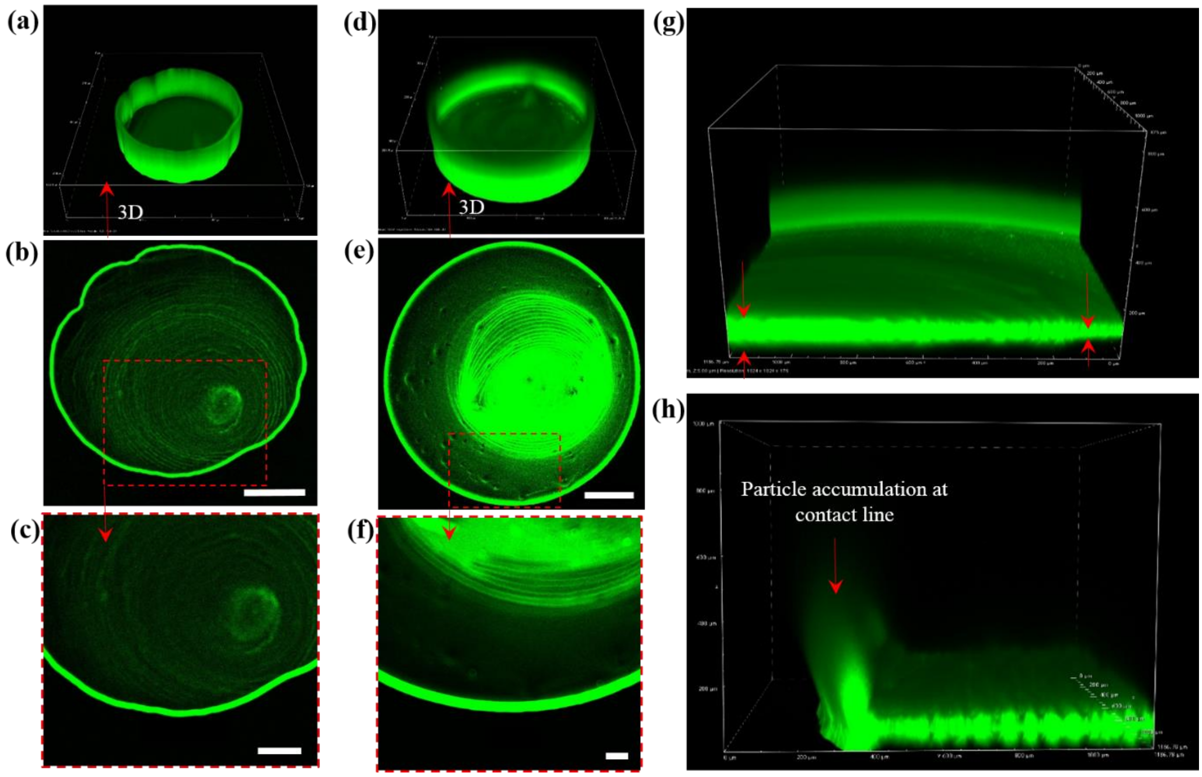

Figure S1. Particle deposition morphologies. **a-c**) Polystyrene microparticle deposition on a glass slide with contact angle= 7°±1°, where a significant percentage (> 80%) of the particles are transported to the contact line; b) scale bar: 1000 µm, c) scale bar: 500 µm. **d-f**) Polystyrene particle deposition on a glass slide with a contact angle of 30°. About 50% of particles migrate to the initial drop contact line while the remaining microparticles form rings as the contact line recedes; e) scale bar=500 µm, f) scale bar=100 µm. **g**) Three-dimensional (3D) image of particle accumulation with increased thickness of the deposited layer in the middle of the wetted area compared to the area close to the perimeter. **h**) 3D representation of the coffee-ring phenomenon illustrating a large portion of particle accumulation at the initial contact line.

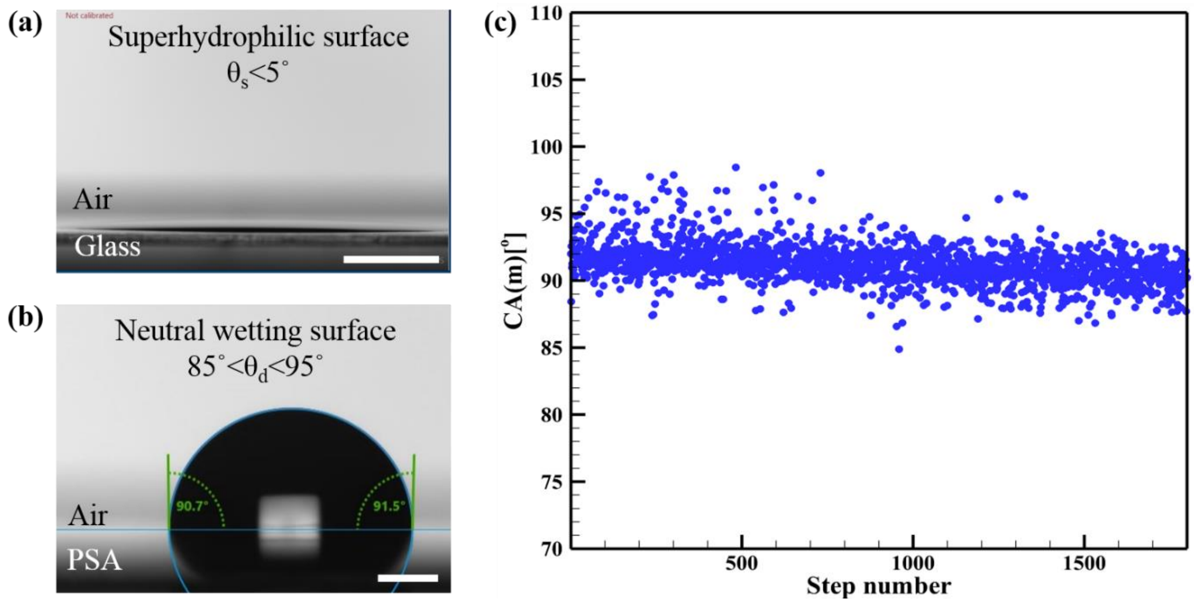

Figure S2. The wettability of the pressure-sensitive adhesive (PSA) and the treated glass slide measured by placing a drop of water on the substrate. **a**) The contact angle of a plasma-treated glass substrate verifies its superhydrophilic surface treatment ($\theta_s < 5\,^{o}$); scale bar: 1000 µm. **b**) Contact angle of a pressure sensitive adhesive (PSA) layer is around 90 ^{o}, confirming the neutral wettability of the PSA; scale bar: 800 µm. **c**) The average contact angle of the PSA with particle-laden liquid, measured over time in different measurement step numbers, shows that the contact angle remains unchanged over time.

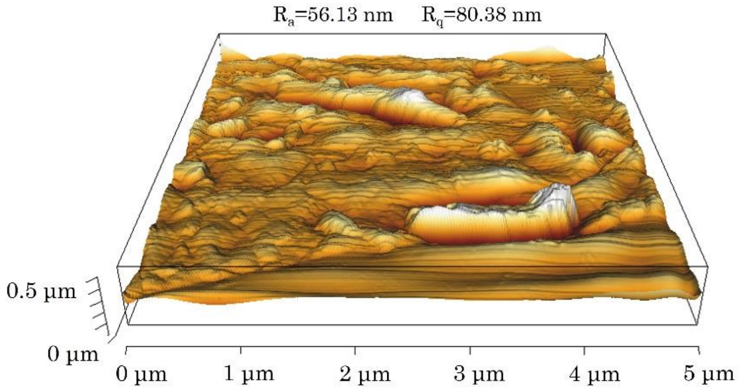

Figure S3. The topography and roughness of the PSA wall, characterized using atomic force microscopy.

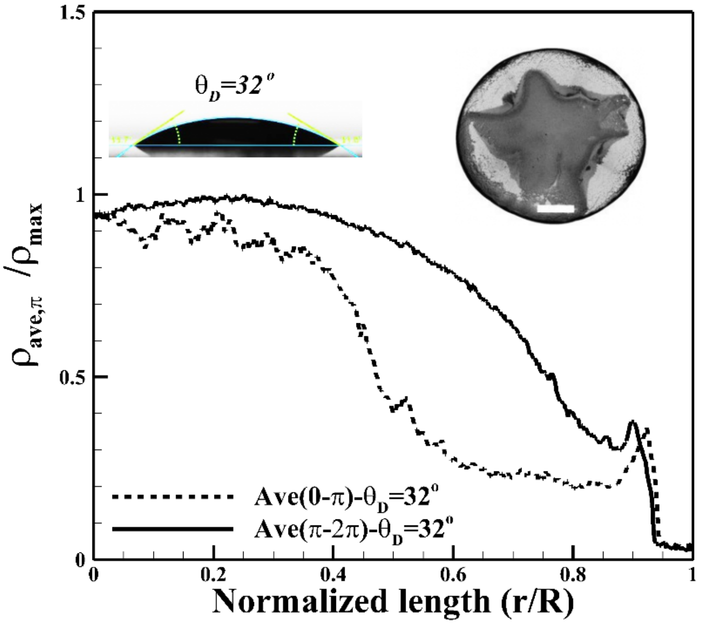

Figure S4. Non-uniform deposition of CNHs nanoparticles on a glass slide with a contact angle of 32°.

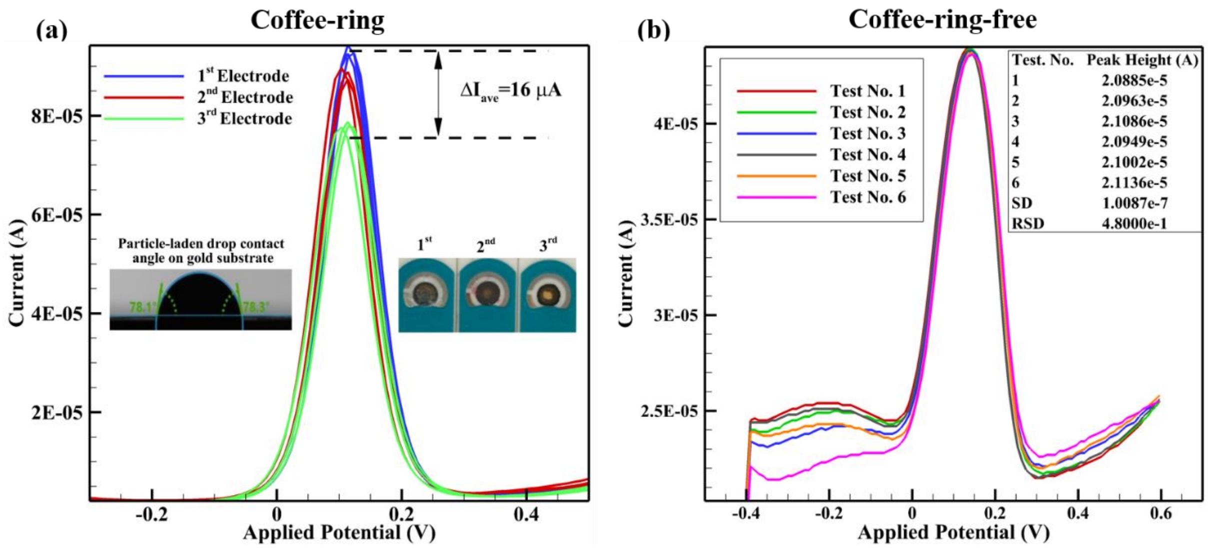

Figure S5. Reproducibility of the CNH particle coatings formed on gold electrodes under coffee-ring and coffee-ring-free conditions, characterized using an electrochemical sensing method. **a**) In the coffee-ring condition, the deposition of CNH particles is not reproducible, indicated by the difference in electrochemical signals. This difference in electrical current among various deposition tests in the coffee-ring condition could reach 16 μA, showing the lack of reproducibility in the coatings (RSD=7.69). **b**) Under the meniscus-free and coffee-ring-free protocol, the successful reproducible deposition of CNHs nanoparticles was characterized using electrochemical sensing (RSD= 0.48).

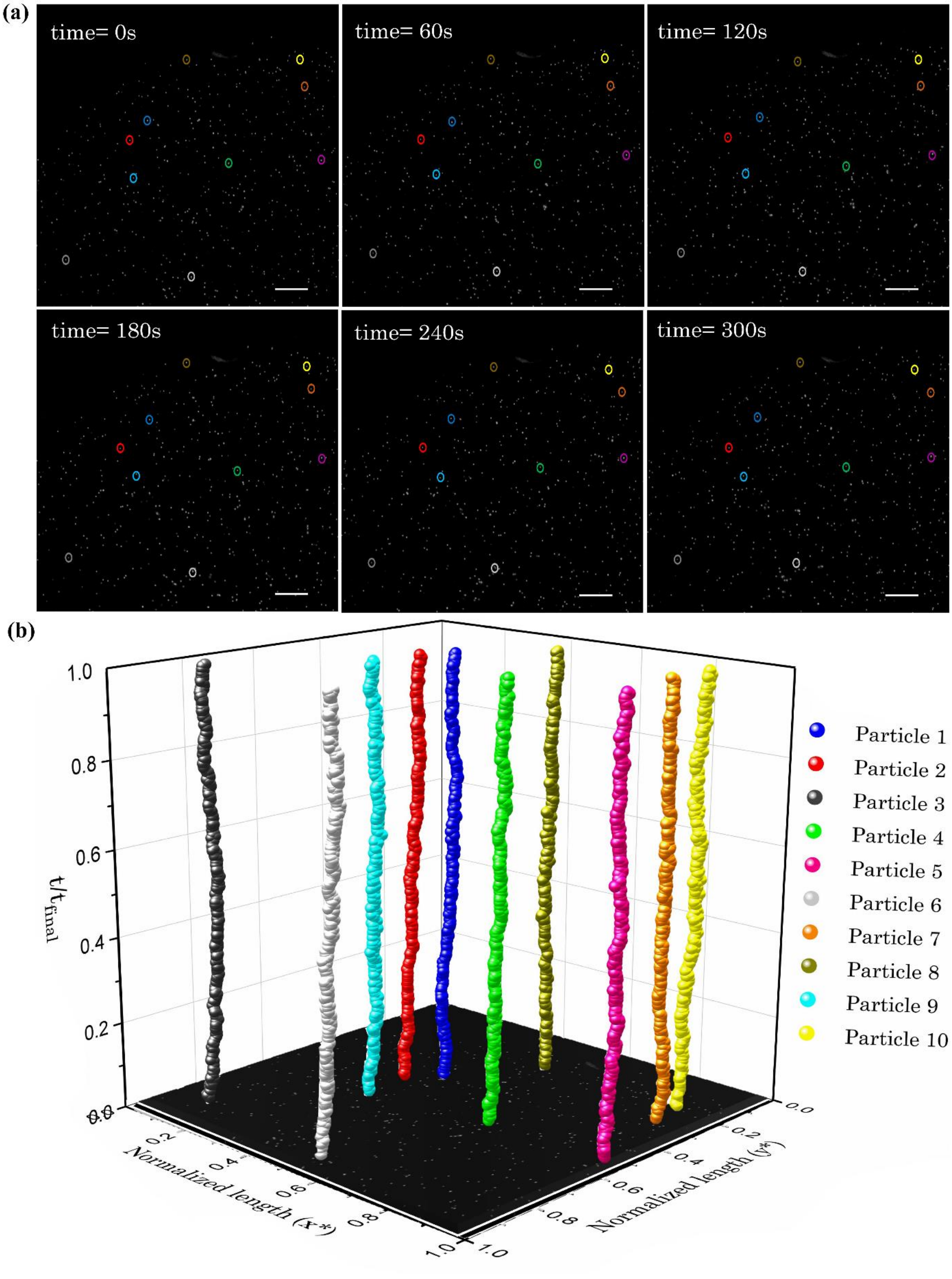

Figure S6. Particle tracing during the liquid film evaporation, characterized using microparticle image velocimetry (μPIV). **a)** Snapshots of the xy position of particles with a size of 1 μm during the film evaporating, representing the zero directional (radial) movements of particles during their precipitation; scale bars: 100 μm. **b)** Pathline of selected particles showing the trajectory of particles during the liquid

film evaporation. The particles follow almost a vertical line trajectory. The particles are monitored at the plane 3 ± 0.5 μm above the substrate. As a thin liquid film is used, the particles can be traced during evaporation, and the vertical motion of the particles does not change the camera focus.

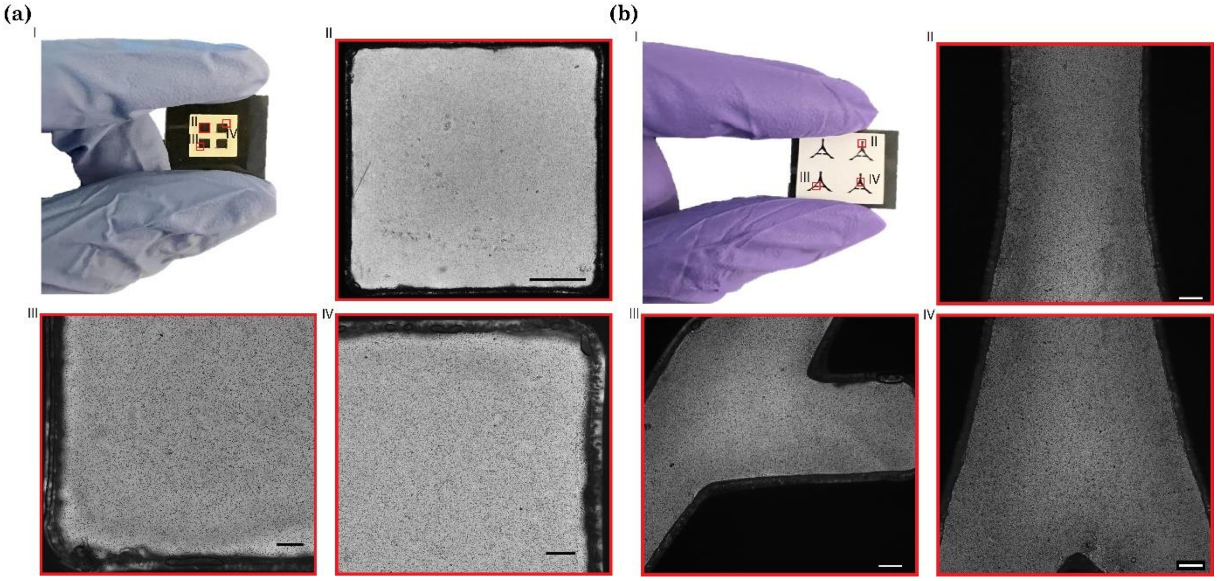

Figure S7. Deposition of CNHs nanoparticles with different mold patterns. Uniform deposition of the particles on **a)** square shape and **b)** A-like shape, representing the negligible effect of the wall curvature on the particle deposition patterns. **a)** Scale bars: subsection II is 500 µm and subsections III and IV are 100 µm. **b)** Scale bars:100 µm.

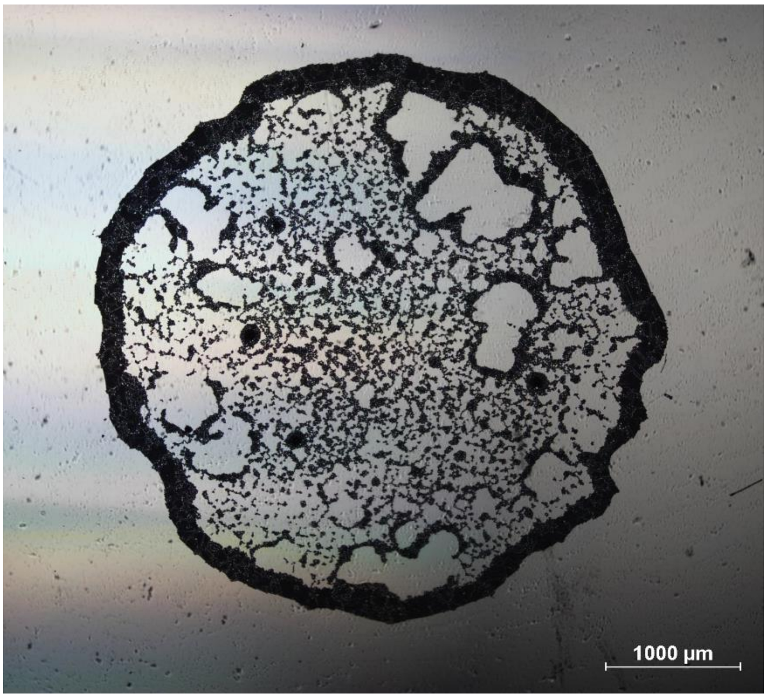

Figure S8. Deposition of 10 µm polystyrene particles on a glass slide. Drop-casting 10 µm particles reveal coffee-ring deposition, confirming that there is a non-uniform deposition of particles even in large particle sizes.

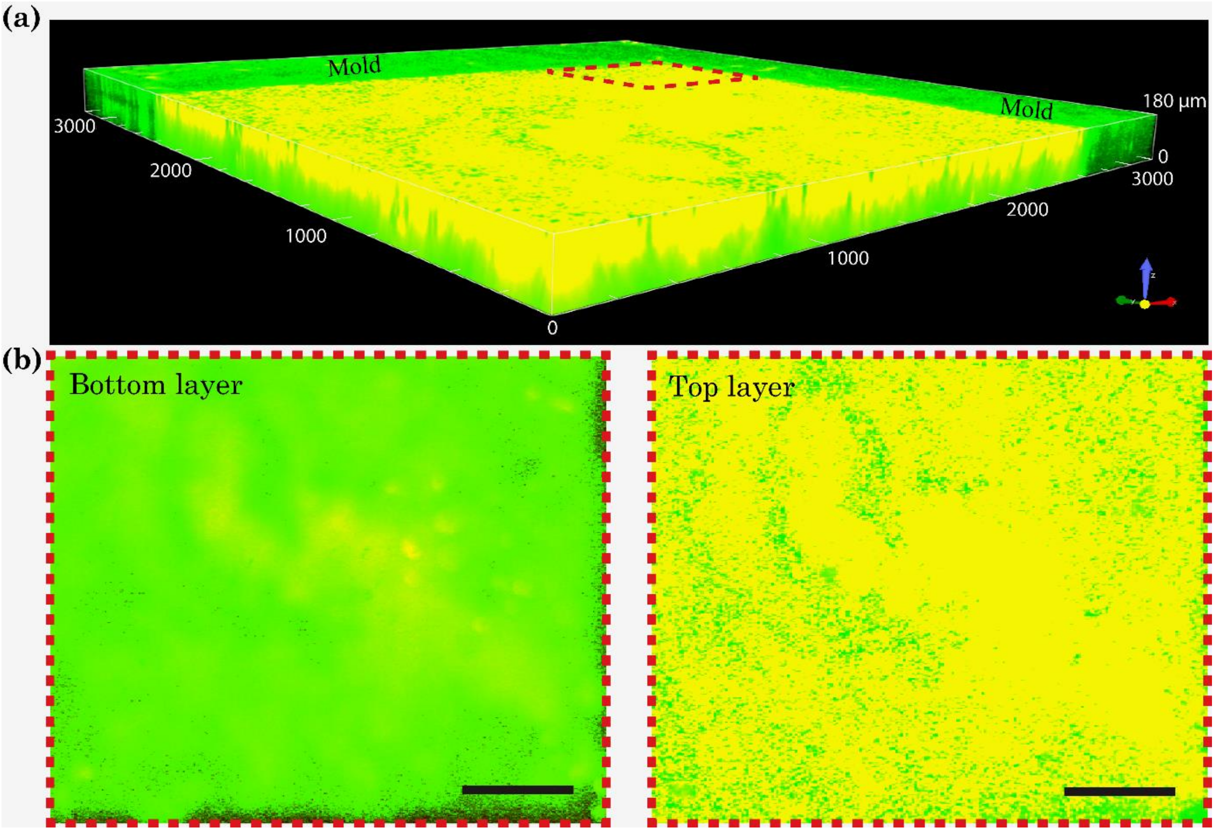

Figure S9. Stratification of polystyrene particles (1000 nm and 200 nm in diameter) at the increased concentration of φ_0= 0.1 (compared to the case with φ_0= 0.01 in Figure 4b) and the initial film thickness of approximately H=500 nm. **a)** 3-dimensional confocal microscopy image of the particles depicts the extended transition zone. **b)** The bottom layer (left) and the top layer (right) of the stratified deposition, shown in Figure S9a, represent the uniform deposition of particles; Scale bars:500 µm.

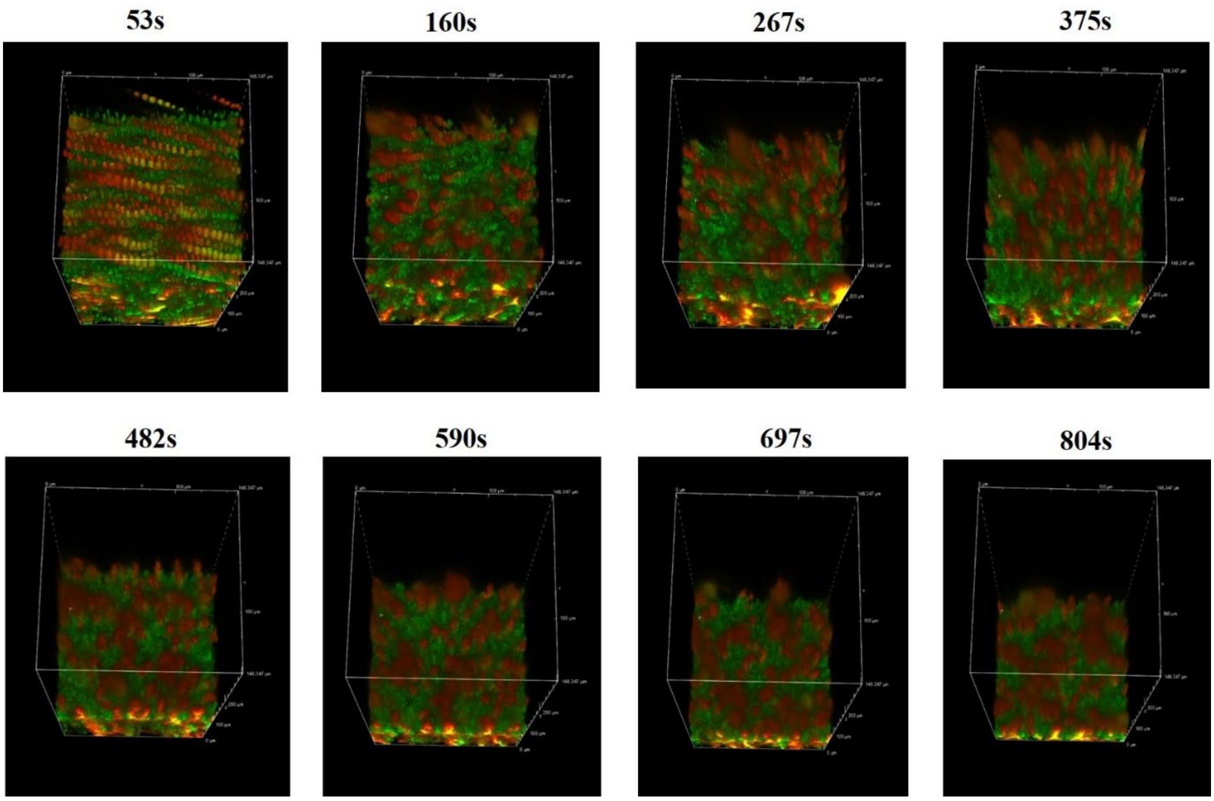

Figure S10. 3D confocal imaging of the auto-organization of mixed fine- and microparticles over time. During evaporation, the red polystyrene microparticles (1000 nm diameter) trap at the descending liquid-air interface while the fine green particles (200 nm diameter) diffuse away from the interface, leading to auto-stratification of these two particle sizes. The stratification in the last deposition steps is minimized because of the high cross interactions among the concentrated particles. The cubic size for all images is 148 µm × 148 µm × 300 µm.

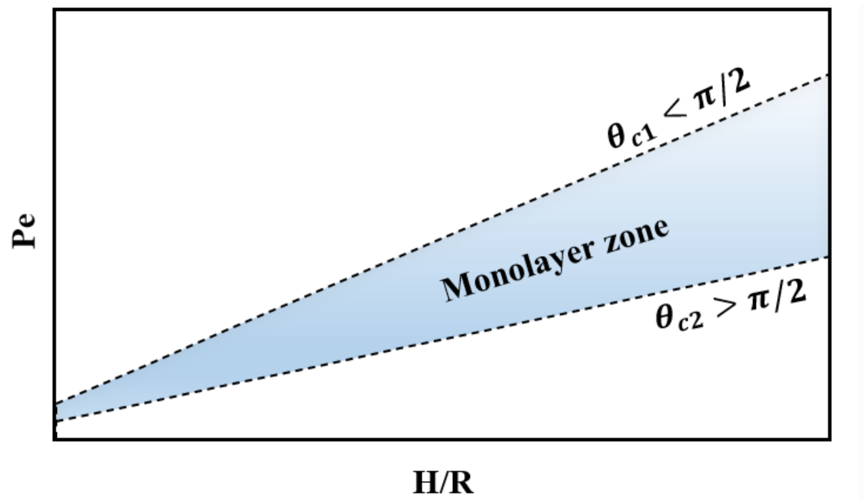

Figure S11. The monolayer zone dependency on Pe and H/R. The zone is narrowing down when H/R decreases as θ_{c1} and θ_{c2} converge to $\theta = \pi/2$, meaning that the control over monolayer deposition in large surface area is higher than the small one. The θ_{c1} and θ_{c2} boundaries also depend on the $|t_{Pin}/\Delta t|$ values.

References

1. Mampallil D, Eral HB. A review on suppression and utilization of the coffee-ring effect. *Advances in Colloid and Interface Science* **252**, 38-54 (2018).

2. Parsa M, Harmand S, Sefiane K. Mechanisms of pattern formation from dried sessile drops. *Advances in Colloid and Interface Science* **254**, 22-47 (2018).

3. Zang D, Tarafdar S, Tarasevich YY, Choudhury MD, Dutta T. Evaporation of a Droplet: From physics to applications. *Physics Reports* **804**, 1-56 (2019).

4. Chen L, Evans JR. Drying of colloidal droplets on superhydrophobic surfaces. *Journal of Colloid and Interface Science* **351**, 283-287 (2010).

5. Brunet P. Particle deposition after droplet evaporation on ultra-hydrophobic micro-textured surfaces. *Soft Matter* **8**, 11294-11301 (2012).

6. Dunn A, *et al.* Laser textured superhydrophobic surfaces and their applications for homogeneous spot deposition. *Applied Surface Science* **365**, 153-159 (2016).

7. Marín ÁG, *et al.* Building microscopic soccer balls with evaporating colloidal fakir drops. *Proceedings of the National Academy of Sciences* **109**, 16455-16458 (2012).

8. Dicuangco M, Dash S, Weibel JA, Garimella SV. Effect of superhydrophobic surface morphology on evaporative deposition patterns. *Applied Physics Letters* **104**, 201604 (2014).

9. Cui L, *et al.* Avoiding coffee ring structure based on hydrophobic silicon pillar arrays during single-drop evaporation. *Soft Matter* **8**, 10448-10456 (2012).

10. Nguyen VX, Stebe KJ. Patterning of small particles by a surfactant-enhanced Marangoni-Bénard instability. *Physical Review Letters* **88**, 164501 (2002).

11. Hu H, Larson RG. Analysis of the effects of Marangoni stresses on the microflow in an evaporating sessile droplet. *Langmuir* **21**, 3972-3980 (2005).

12. Majumder M, *et al.* Overcoming the "coffee-stain" effect by compositional Marangoni-flow-assisted drop-drying. *The Journal of Physical Chemistry B* **116**, 6536-6542 (2012).

13. Ta V, *et al.* Dynamically controlled deposition of colloidal nanoparticle suspension in evaporating drops using laser radiation. *Soft Matter* **12**, 4530-4536 (2016).

14. Parsa M, Harmand S, Sefiane K, Bigerelle M, Deltombe R. Effect of substrate temperature on pattern formation of nanoparticles from volatile drops. *Langmuir* **31**, 3354-3367 (2015).

15. Lim JA, Lee WH, Lee HS, Lee JH, Park YD, Cho K. Self‐organization of ink‐jet‐printed triisopropylsilylethynyl pentacene via evaporation‐induced flows in a drying droplet. *Advanced Functional Materials* **18**, 229-234 (2008).

16. Sempels W, De Dier R, Mizuno H, Hofkens J, Vermant J. Auto-production of biosurfactants reverses the coffee ring effect in a bacterial system. *Nature Communications* **4**, 1-8 (2013).

17. Still T, Yunker PJ, Yodh AG. Surfactant-induced Marangoni eddies alter the coffee-rings of evaporating colloidal drops. *Langmuir* **28**, 4984-4988 (2012).

18. Erbil HY. Control of stain geometry by drop evaporation of surfactant containing dispersions. *Advances in Colloid and Interface Science* **222**, 275-290 (2015).

19. Seo C, Jang D, Chae J, Shin S. Altering the coffee-ring effect by adding a surfactant-like viscous polymer solution. *Scientific Reports* **7**, 1-9 (2017).

20. Kim H, Boulogne F, Um E, Jacobi I, Button E, Stone HA. Controlled uniform coating from the interplay of Marangoni flows and surface-adsorbed macromolecules. *Physical Review Letters* **116**, 124501 (2016).

21. Kim SJ, Kang KH, Lee J-G, Kang IS, Yoon BJ. Control of particle-deposition pattern in a sessile droplet by using radial electroosmotic flow. *Analytical Chemistry* **78**, 5192-5197 (2006).

22. Das S, Chakraborty S, Mitra SK. Ring stains in the presence of electrokinetic interactions. *Physical Review E* **85**, 046311 (2012).

23. Mampallil D, Eral H, Van Den Ende D, Mugele F. Control of evaporating complex fluids through electrowetting. *Soft Matter* **8**, 10614-10617 (2012).

24. Yunker PJ, Still T, Lohr MA, Yodh A. Suppression of the coffee-ring effect by shape-dependent capillary interactions. *Nature* **476**, 308-311 (2011).

25. Bhardwaj R, Fang X, Somasundaran P, Attinger D. Self-assembly of colloidal particles from evaporating droplets: role of DLVO interactions and proposition of a phase diagram. *Langmuir* **26**, 7833-7842 (2010).

26. Harris DJ, Hu H, Conrad JC, Lewis JA. Patterning colloidal films via evaporative lithography. *Physical Review Letters* **98**, 148301 (2007).

27. Lin Z, Granick S. Patterns formed by droplet evaporation from a restricted geometry. *Journal of the American Chemical Society* **127**, 2816-2817 (2005).

28. Hong SW, Byun M, Lin Z. Robust Self‐Assembly of Highly Ordered Complex Structures by Controlled Evaporation of Confined Microfluids. *Angewandte Chemie International Edition* **48**, 512-516 (2009).

29. Al‐Milaji KN, Secondo RR, Ng TN, Kinsey N, Zhao H. Interfacial Self‐Assembly of Colloidal Nanoparticles in Dual‐Droplet Inkjet Printing. *Advanced Materials Interfaces* **5**, 1701561 (2018).

30. Al-Milaji KN, Radhakrishnan V, Kamerkar P, Zhao H. pH-modulated self-assembly of colloidal nanoparticles in a dual-droplet inkjet printing process. *Journal of Colloid and Interface Science* **529**, 234-242 (2018).

31. Ko H-Y, Park J, Shin H, Moon J. Rapid self-assembly of monodisperse colloidal spheres in an ink-jet printed droplet. *Chemistry of Materials* **16**, 4212-4215 (2004).

32. Li Y, Diddens C, Segers T, Wijshoff H, Versluis M, Lohse D. Evaporating droplets on oil-wetted surfaces: Suppression of the coffee-stain effect. *Proceedings of the National Academy of Sciences* **117**, 16756-16763 (2020).

33. Tian D, Song Y, Jiang L. Patterning of controllable surface wettability for printing techniques. *Chemical Society Reviews* **42**, 5184-5209 (2013).

34. Young T. III. An essay on the cohesion of fluids. *Philosophical transactions of the royal society of London*, 65-87 (1805).
