# Supplementary material for: Self-assembly of highly ordered micro- and nanoparticle deposits
Source: Nat Commun. 2022 Jun 2;13:3085. doi: 10.1038/s41467-022-30660-6 (PMC9163176; doi:10.1038/s41467-022-30660-6)
Supplement: Supplementary file 3 — Description of Additional Supplementary Files [file 41467_2022_30660_MOESM3_ESM.pdf]

Description of Additional Supplementary Files

File Name: Supplementary Movie 1
Description: Coffee ring formation-30x.mp4
The migration of carbon nanohorn particles to the pinned contact line during drop evaporation on an untreated glass slide forming a coffee-ring pattern.

File Name: Supplementary Movie 2
Description: coffee-ring-free-40x.mp4
The uniform deposition of the particles using meniscus-free and coffee-ring-free method. The particles represent no outward/inward movement due to eliminating the drop curvature.

File Name: Supplementary Movie 3
Description: Amorphous particle accumulation.mp4
The three-dimensional confocal microscopy movie of the fluorescent particles shows an amorphous accumulation of particles at the contact line when the coffee-ring phenomenon occurs.

File Name: Supplementary Movie 4
Description: Stratification of particles-Uniform coating.mp4
The three-dimensional confocal microscopy movie of the deposited mixed fluorescent particles with two different sizes (1000 nm and 200 nm) using the proposed method. It represents the self-stratification and uniform coating of the particles in the designed pattern.

File Name: Supplementary Movie 5
Description: μPIV-portion of circle-2x.mp4
Tracing the movement of particles (1000 nm in diameter) during the liquid film evaporation in a circular geometry, using μPIV analysis. It represents no radial movement of particles during film evaporation under the MFCF method.

File Name: Supplementary Movie 6
Description: μPIV-portion of square-2x.mp4
Tracing the movement of particles (1000 nm in diameter) during the liquid film evaporation in a rectangular geometry using μPIV analysis. It represents no edgeward movement of particles, due to wall non-circular curvature, during film evaporation under the proposed method.
